# Gamma Delta T Cells and Their Involvement in COVID-19 Virus Infections

**DOI:** 10.3389/fimmu.2021.741218

**Published:** 2021-10-29

**Authors:** Georg von Massow, Steve Oh, Alan Lam, Kenth Gustafsson

**Affiliations:** ^1^ Department of Biochemical Engineering, University College London, London, United Kingdom; ^2^ Bioprocessing Technology Institute, A*STAR, Singapore, Singapore

**Keywords:** COVID-19, gamma-delta T cell, SARS-Cov-2, lymphopenia, T cells

## Abstract

The global outbreak of the SARS-Cov-2 virus in 2020 has killed millions of people worldwide and forced large parts of the world into lockdowns. While multiple vaccine programs are starting to immunize the global population, there is no direct cure for COVID-19, the disease caused by the SARS-Cov-2 infection. A common symptom in patients is a decrease in T cells, called lymphopenia. It is as of yet unclear what the exact role of T cells are in the immune response to COVID-19. The research so far has mainly focused on the involvement of classical αβ T cells. However, another subset of T cells called γδ T cells could have an important role to play. As part of the innate immune system, γδ T cells respond to inflammation and stressed or infected cells. The γδ T cell subset appears to be particularly affected by lymphopenia in COVID-19 patients and commonly express activation and exhaustion markers. Particularly in children, this subset of T cells seems to be most affected. This is interesting and relevant because γδ T cells are more prominent and active in early life. Their specific involvement in this group of patients could indicate a significant role for γδ T cells in this disease. Furthermore, they seem to be involved in other viral infections and were able to kill SARS infected cells *in vitro*. γδ T cells can take up, process and present antigens from microbes and human cells. As *e.g.* tumour-associated antigens are presented by MHC on γδ T cells to classical T-cells, we argue here that it stands to reason that also viral antigens, such as SARS-Cov-2-derived peptides, can be presented in the same way. γδ T cells are already used for medical purposes in oncology and have potential in cancer therapy. As γδ T cells are not necessarily able to distinguish between a transformed and a virally infected cell it could therefore be of great interest to investigate further the relationship between COVID-19 and γδ T cells.

## Introduction

The SARS-Cov-2 virus developed into a worldwide pandemic in a matter of months. A year after its first reporting it has infected over 121.256.160 people and killed 2.681.790 worldwide (Hopkins University 18.03.2021). Because of this, the scientific community has been researching the development of potential cures and vaccines to stop the pandemic. This has led to the development of multiple vaccines (by e.g. Pfizer/BioNTech, Moderna and AstraZeneca).

Never before in history has so much scientific effort been put towards one single illness. Thanks to this, a lot is already known about this disease. There has been a lot of research focusing on how the virus works and how our immune system tries to deal with the disease it causes. Yet, there is no direct cure for it. This might be because there are still certain gaps in our knowledge of how our immune system copes with COVID-19.

Recently, the discovery of multiple new variants in the UK, South Africa and Brazil have given even more reason to find a cure (1, 2). These variants seem to differ in their infectivity, which could make them an even bigger threat than the original variant (3).

There is a lot of research focusing on the involvement of adaptive immunity. The adaptive immune system develops over a human’s life span and can, as the name suggests, adapt to new arising challenges. The αβ T cells, which are the hallmark T lymphocytes representing adaptive immunity, have been extensively studied in relation to COVID-19 (4, 5) over this short time-frame. However, another part of the immune system also shows a lot of promise in the fight against COVID-19. The innate immune system is present since birth and defends us against a plethora of diseases and illnesses (6). While it doesn’t adapt to new challenges in an individual, it has evolved to respond to a large variety of challenges. γδ T cells make up a part of the innate immune system (7).

**Figure 1:**
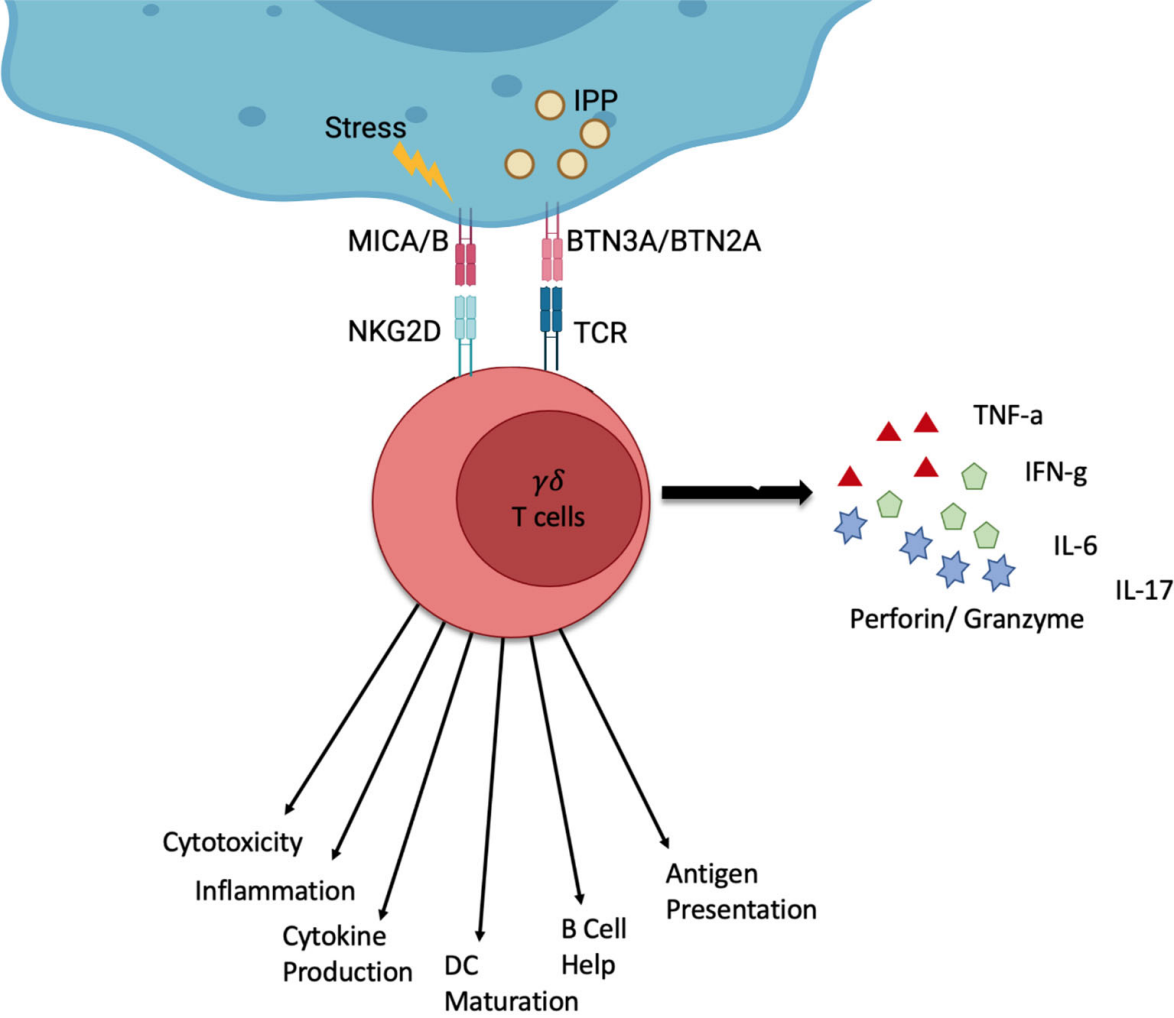
Schematic of the function of γδ T cells. γδ T cells can be activated by stress signals from infected or tumour cells. Stressed or infected cells can express MICA and MICB which γδ T cells can recognise *via* the NKG2D receptor. Furthermore, the overexpression of certain molecules such as IPP can be recognised through the γδ TCR by recognising BTN3A and BTN2A on stressed or infected cells. γδ T cells can respond *via* different channels. This can be the production of cytokines (IFN-γ, TNF-α, IL-6, IL-17) or cytolysis (Perforin, Granzyme). Additionally, γδ T cells can interact with other immune cells and present antigens. Created by BioRender.

These T cells are characterised by their T cell receptor (TCR), which consists of a γ- and a δ-chain. These immune cells use their TCR to recognise a host of signals. γδ T cells are particularly interesting for cell therapy because, unlike their αβ T cell counterparts, they are major histocompatibility complex (MHC) independent (8, 9). Their TCR can recognise stress signals from infected or tumour cells. The TCR of Vγ9Vδ2 T cells, the most common subtype in peripheral blood, binds isopentenyl pyrophosphate (IPP) which is an intermediate in the mevalonate pathway and often overexpressed in cancer cells (9) (**Figure 1**) as well as in infected cells.

They have, therefore, been the focus of cancer research development of novel cell therapies (10). Their MHC independence makes them especially attractive for allogeneic T-cell treatment. This also makes them interesting for COVID-19 treatments.

γδ T cells can perform a number of functions, which include the production and secretion of cytokines, such as IFN- γ, TNF-α, IL-6 and IL-17. They can also perform cytolysis through cytotoxic perforin and granzyme actions as well as interact with other immune cells like B cells and dendritic cells (11). Vγ9Vδ2 T cells, in particular, are also capable of presenting peptide antigens to classical T-cells in a ‘professional’ fashion (12, 13).

More recently our lab and others were able to show that the Vγ9Vδ2 T cell subset is able to phagocytose certain bacteria and parasite infected cells such as *E. coli* and *P falciparum* (14, 15). These cells were no longer predominantly inflammatory cytotoxic cells but, professional phagocytic cells. With the phagocytosis capability also comes the ability to professionally present antigens.

This discovery further highlights the potential importance of γδ T cells in the early stages of an infection and their versatility. Their ability to phagocytose and act as antigen-presenting cells is a new discovery and needs to be further explored. However, this could become a highly relevant topic in viral infections such as SARS-CoV 2 as well.

In this review we bring together what is so far known about the link between COVID-19 and γδ T cells. To understand the relevance of this link we will give a brief overview of cytokine release syndrome (CRS) or ‘cytokine storm’ and fibrosis, which are two common life-threatening complications in COVID-19 patients. Then we will briefly summarize the current understanding of the response of T cells in general to COVID-19. Finally, we review the current research into γδ T cells in COVID-19 as well as γδ T cells as potential cell therapies in a viral infectious disease context.

## Cytokine Storm and Fibrosis

A Cytokine Release Syndrome (CRS) or ‘cytokine storm’ is a hyperactivation of the immune system through cytokines, causing high fever, lymphopenia and in severe cases acute respiratory distress syndrome (16, 17). However, the pathogenesis is still not fully defined (18).

It has been reported in patients of various diseases and infections and has been observed in MERS and SARS patients and more recently in SARS-Cov-2 infections (19, 20). Cytokine storm is caused by an uncontrolled host immune reaction which causes an activation cascade of cytokine production that are auto-amplifying (21). However, the causes can be varied, and cytokine storms are defined more by the final clinical phenotype, rather than the initiating factors. The clinical phenotype is a systemic inflammation, multi organ-failure and it can lead to death if left untreated.

Interestingly, the cytokines in the cytokine storm can be both pro-inflammatory and immunosuppressive (22). IL-10 is a prominent immunosuppressive cytokine that indicates a shift away from the cytokine storm towards a form of “immunoparalysis” (18, 23). This downregulation of systemic immunological functions seems to be beneficial in regulating systemic reactions to local infection (24). However, prolonged immunoparalysis after cytokine storm and severe sepsis can lead to complications and an increase in mortality in the if not reversed (25).

The proinflammatory cytokines that cause the cytokine storm can vary. In the case of SARS those are IL-6, interferons, particularly IFN- γ, and IL-1β, among others (26). In SARS these cytokines seem to be produced in large quantities by infected macrophages and dendritic cells (27–29). The infection of these cells is abortive in SARS, however in COVID-19 patients infected CD169^+^ macrophages have caused damage to the lymphoid tissue (30). These macrophages cause activation-induced-cell-death by expressing high levels of Fas. Furthermore, these infected macrophages appear to promote lymphocyte necrosis through the production of IL-6 by signalling through the STAT3 pathway (30). The lymphocyte levels could be partially restored after patients were given a Tocilizumab treatment, which is an IL-6 inhibitor which further supports that lymphopenia is at least in part caused by infected macrophages in COVID-19 patients (31). However, the use of Tocilizumab on its own was not able to improve the survival rate of Covid-19 patients (32, 33).

In Covid-19, IL-6, together with IL-10 seem to be the only cytokines consistently elevated among reports, while TNF-α is elevated in some reports (31, 34, 35). The elevation of these cytokines seems to correlate with the decrease of the total T cell levels (34).

While T cells produce cytokines themselves, it appears that the inducers of the cytokine storm in COVID-19 seem to be infected macrophages and dendritic cells that start the cascade. This could give hope, that medical treatments aimed at increasing certain T cell levels might aid in recovery. This is especially intriguing when considering a combination of IL-6 inhibitors to suppress the immunosuppressive capacities of this cytokine.

Another alarming finding in SARS-Cov-2 infected patients is pulmonary fibrosis. Fibrosis is the scaring or damaging of organ tissue. In the case of pulmonary fibrosis, the scaring and damage occurs to the lungs and is irreversible. There have now been multiple reports of this type of fibrosis affecting Covid-19 survivors (36–38). This means that even after a patient survives the disease, they might have to deal with the consequences the rest of their life.

Interestingly, there is research suggesting that IL-17 is involved in the development of fibrosis in murine models (39, 40). This is further supported in findings in different types of human fibrosis, like hepatitis-B-related fibrosis or cystic fibrosis (41–43).

This in turn is relevant in a γδ T cell context because γδ T cells are a producer of IL-17 (44, 45). It is important to note that other immune cells such as Th17 cells, which are an αβ T cell subset, are also producers of IL-17 (46). However, Th17 cells require antigen-specific priming to do so. In the case of γδ T cells there are both natural and inducible IL-17 producing cells called Tγδ17 cells (47). The natural Tγδ17 cells are considered to be localised in the peripheral mucosal tissues and in mice have been found in lung tissues (48–52). These natural γδ T cells can respond quickly to infection and are found to produce IL-17 within 24h (48, 53).

The inducible Tγδ17 cells seem to mature and differentiate in lymph nodes in order to make IL-17 in an immune response after encountering antigens (54). These γδ T cells seem to produce IL-17 within 60h post antigen contact.

There is not yet any indication that these subtypes are directly involved in COVID-19. Especially, when considering that there are a number of immune cells that can produce IL-17 (46). Nevertheless, γδ T cell have the unique ability to both muster an innate and quick IL-17 response as well as a specific antigen dependent IL-17 response to an infection.

It is therefore important to look at this Covid-19 complication with γδ T cells in mind; especially, when considering that IL-17 has been found to be elevated in Covid-19 patients (55). Jouan and colleagues, further found, that the IL-17 was more concentrated in the lung tissue when compared to blood levels. This gives further indication of a link between Covid-19 caused fibrosis and IL-17.

γδ T cells could thus play a considerable role in Covid-19 symptoms. In both the case of cytokine storm as well as fibrosis they might affect the patients in a negative way. It is therefore important to look at potential γδ T cell based approaches with potential side effects in mind. In the case of fibrosis, IL-17 inhibitors such as Secukinumab could be combined with a γδ T cell based therapy.

## T Cells and COVID-19

The relationship between COVID-19 and αβ T cells has already been reviewed elsewhere (56). However, we want to highlight a few key points that might be relevant for γδ T cells. Lymphopenia seems to be a common symptom among COVID-19 patients (5, 31, 57–59). But the T cell levels appear to recover after the patients overcome the illness (5, 60). Lymphopenia is common in respiratory viral infections (61) In Covid-19, while the lymphopenia is consistent in the CD4^+^ T cell subtype across reports, the severity of lymphopenia in the CD8^+^ subtype varies (62, 63).

Potential causes for this lymphopenia could be hyperactivation or exhaustion. This is supported by the findings of activation markers and pro-apoptotic molecules (35, 57). The CD8^+^ subtype seems to be activated to a larger degree compared to the CD4^+^ subtype. However, it isn’t clear if these cytotoxic CD8^+^ T cells are hyperactive or exhausted, which would affect their involvement in COVID-19. Some research suggests hyperactivated CD8^+^ T cells (64), while other research suggests exhausted CD8^+^ T cells with reduced cytokine production (65).

Interestingly, studies have found memory CD4^+^ and CD8^+^ T cells in recovered patients that are virus-specific (4, 63, 66). It is not yet determined if these memory T cells in fact, provide immunity to survivors.

All this indicates an involvement of αβ T cells in COVID-19. However, to what extent they aid in combating the illness is not certain. There does appear to be a large variance between patients, which calls for distinct therapy approaches based on the patients differing T cell profiles.

## γδ T Cells and COVID-19

So far, the research into T cell involvement as mentioned above, has quite heavily focused on the αβ T cells. Therefore, there is limited information on the involvement of γδ T cells. However, what we do know so far can give a good indication of the relevance of γδ T cells.

First of all, the Lymphopenia that is reported doesn’t exclusively affect αβ T cells, but seems to affect γδ T cells just as much (67–70) (**Table 1**). Intriguingly, one study showed that patients with the most severity had the lowest amount of γδ T cells (67).

**Table 1:** Studies reporting on the effects of Covid-19 on γδ T cells and related cytokines.

	Donor Cohort	Covid-19 Patients	Blood *vs* Tissue	Ref
γδ T cells	98 healthy controls, 7 healthy child controls, 25 child patients, 68 patients	Decreased	Not significant	(55, 68–70)
δ2 T cells	92 healthy controls, 84 adult patients	Decreased	NA	(55, 67, 70)
δ1 T cells	20 healthy controls, 30 adult patients	No significant difference	NA	(55)
Activation Markers	38 healthy controls, 68 patients	Increased	NA	(55, 68)
Exhaustion Markers	20 healthy controls, 30 patients	increased	Higher in Tissue than in Blood	(55)
IFN-γ	20 healthy controls, 7 healthy child controls, 30 patients, 23 child patients	No significant difference	Higher in Tissue than in Blood	(55, 69)

NA, not applicable.

This begs the question, if γδ T cell levels in patients could be indication of the severity of the illness. It is however not clear how the level of γδ T cells in a patient before and at the beginning of the disease impacts the outcome.

There also seems to be a significant shift in the phenotype of γδ T cells in patients. There are conflicting reports regarding these changes. One study suggests a transition to a naïve phenotype both in percentage as well as in absolute numbers (n=30) (70). This shift does not seem to be affected by the severity of the disease. In contrast, in another study with significantly less patients (n=5) a shift towards an effector (memory) phenotype was detected over the span of two weeks.

**Figure 2:**
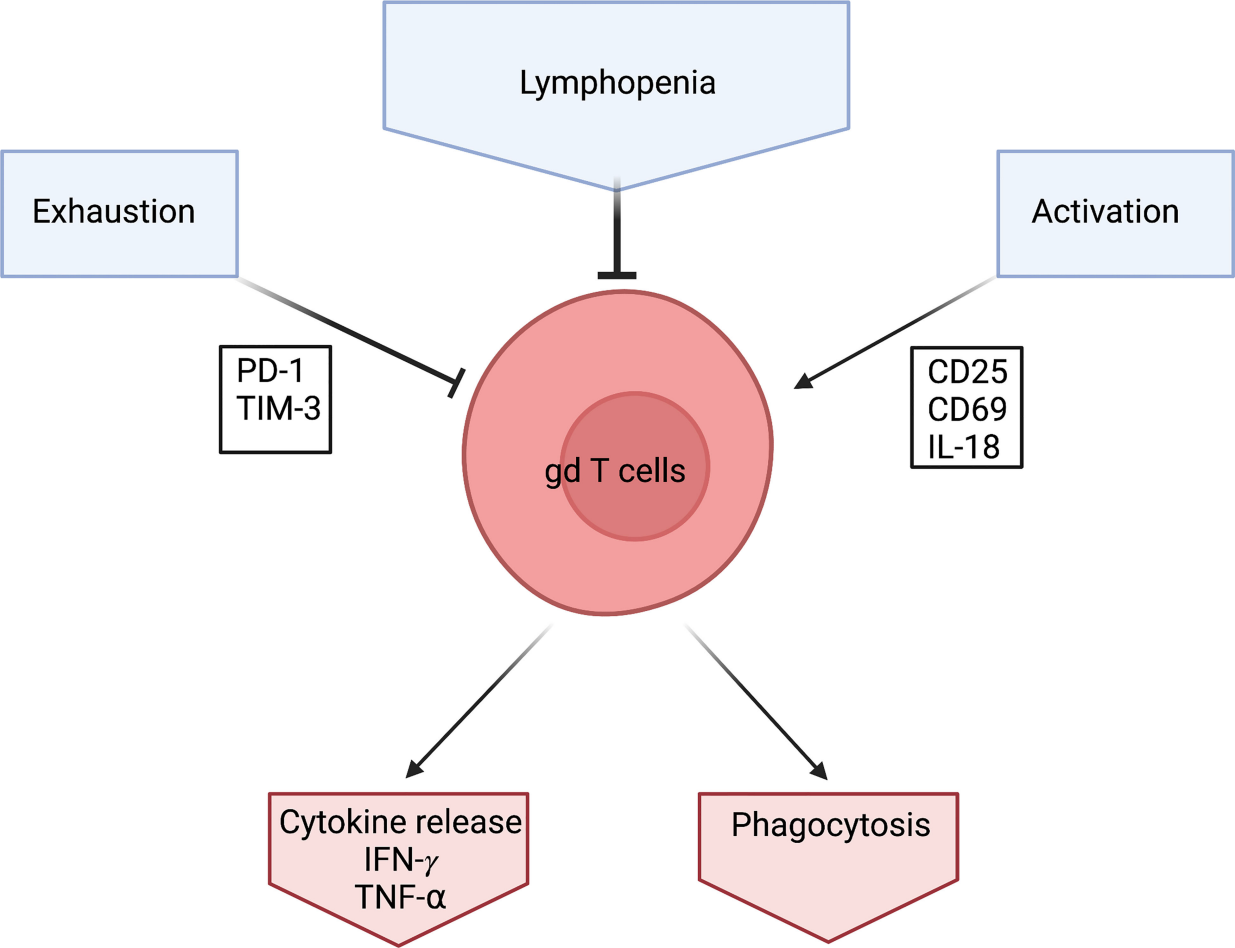
Schematic of what effects γδ T cells during a Covid-19 infection. γδ T cells levels are low in Covid-19 patients which is characterized by Lymphopenia. They seem to be exhausted which is detected *via* the exhaustion marker PD-1. They also seem to be heavily activated which is shown through activation markers CD25, CD69 and IL-18. However, γδ T cells produce high amounts of IFN- γ in response to Covid-19. Created by BioRender.

A transition to the naïve phenotype seems counterintuitive. The reports of lymphopenia would suggest an activation and exhaustion of γδ T cells similar to that reported in αβ T cells (see above) and therefore a reduction of the naïve phenotype in the overall population. This is also supported by the upregulation of the activation marker CD25 in COVID 19 patients (68). and further supported by high expression levels of CD69 and PD-1 on γδ T cells (55). CD69 is an early activation marker and PD-1 is an exhaustion marker for some T cells (71, 72) (**Figure 2**).

The role of PD-1 in the context of γδ T cells is as of yet not fully understood. For CD4 & CD8 αβ T cells PD-1 upregulation seems to indicate exhaustion (73). However, this doesn’t seem to apply to all T cells; for example, follicular T helper cells seem to highly express PD-1 and PD-1 seems to be more involved in tissue position control (74). More importantly there are now reports indicating that PD-1 expressing SARS-CoV2 CD8^+^ αβ T cells are functional and not exhausted (75).

The role of PD-1 expression for γδ T cells is not fully known yet. On the one hand, tumour-bearing mice derived γδ T cells that were PD-1^+^ showed signs of exhaustion and apoptosis (76). On the other hand, studies on δ2 T cells show that the relationship is more complex. The cytotoxic activity of PD-1^+^ and PD-1^-^ γδ T cells were comparable against zoledronate treated PD-1^+^ Daudi cells (77). This would indicate that γδ T cells could overcome the inhibitory effect of PD-1 through phosphoantigen stimulated TCR triggering.

In a more recent study in acute myeloid leukemia patients, the exhaustion of δ2 T cells seemed to be more indicated by TIM-3 (78). In fact, the expression of TNF-α and IFN-γ was highest in the PD-1^+^ TIM-3^-^ subset and lowest in the double negative subset. A role of TIM-3 in γδ T cell exhaustion was also shown in malaria patients that showed high expression of TIM-3 was linked to reduced δ2 T cell pro-inflammatory cytokine production (79).

The meaning of high expression of PD-1 on γδ T cells in COVID-19 patient needs further investigation. It does indicate an involvement of γδ T cells in the immune response but other exhaustion markers, other than TIM-3, need to be investigated to examine how involved the γδ T cell response in COVID-19 is.

The involvement of γδ T cells in COVID-19 is further supported, by an increase in IL-18, reported in the same study by Jouan et al. IL-18 is a cytokine that seems to be involved in γδ T cell activation in viral infections (80).

Considering all these different activation and exhaustion markers being expressed in COVID-19 patients, it appears paradoxical to concomitantly have a significant increase in both relative as well as absolute naïve γδ T cells levels.

However, most of these reports are based on blood samples from patients. This neglects the fact that there are major differences between the circulating γδ T cells and those recruited into the inflamed tissues (81). In general, the relative levels of the γδ T cell subtypes seems to differ between the blood stream and tissues. The δ1 subtype is more frequently encountered in tissues, whereas the δ2 subtype is mostly found in the blood in the form of Vγ9Vδ2 T cells (82–84). In the case of an infection, more γδ T cells would be recruited into the tissue and it is therefore of interest to look at the differences of blood and tissue T cell levels.

So far there has only been one study that focuses on the differences between blood and tissue in relation to γδ T cells and COVID-19 (55). In this study blood samples were compared with endotracheal aspirates (n=30). Interestingly, they did not find any differences in the γδ T cell levels between blood and tissue in COVID-19 patients. It was also not clear if the subtypes changed between blood and tissue during the disease. However, not only do they find an increase of the activation marker CD69 and the exhaustion marker PD-1 between healthy controls and patients, but they find a significant increase of these factors in the tissue compared to the blood. While they do not analyse the phenotype of the tissue residing γδ T cells, the activation markers would indicate a high level of activation of these γδ T cells.

The differences in tissue *vs* blood could be a great opportunity for further research. γδ T cells are a very diverse group of immune cells and differ greatly in function, especially between tissue and circulating γδ T cells (85). So far circulating γδ T cells have been studied the most with the assumption that they infiltrate tissue when infected. However, the tissue residing γδ T cell subtypes have been studied less, although they are potentially the first responders to tissue infections.

Tissue residing γδ T cells are generally δ1 T cells (or with other δ chains, such as δ3) while circulating γδ T cells are usually δ2 T cells which means their TCR is built differently, with either a δ1 or δ2 chain. This is potentially relevant for their functions, not least of which the recently discovered ability to phagocytose infected cells and professionally present antigens in a TCR-dependant manner (14, 15). This has so far only been studied with Vγ9Vδ2 T cells. It would be of relevance to study, if different γδ T cells with different TCRs can show similar functions. Furthermore, tumour-associated antigens can be taken up by γδ T cells and presented to classical T-cells by MHC molecules in a professional capacity reminiscent of dendritic cells (DC) (86, 87). It therefore stands to reason that also virally infected cells, similarly killed by a SARS virus (88), should be able to provide viral peptides for MHC processing and cross-presentation. If so, this may have profound consequences for the immunology of SARS-Cov-2 and for improved vaccine strategies. In support of this strong possibility it has been shown that γδ T cells can orchestrate specific CD8+ T-cell responses to Epstein Barr virus epitopes (89).

It is important to note, that not all functions of γδ T cells require their TCR, for example γδ T cell can target cancer cells through the NKG2D receptor, independently of the TCR (90).

COVID-19 patients show elevated IFN- γ levels (69) and Jouan et al. (55) could show that there was a significant increase in the tissue compared to blood. The amount of IFN- γ producing innate immune cells in the blood, including γδ T cells were reduced in patients. This would indicate that there could be a higher amount of IFN- γ producing γδ T cells in the tissue to generate the high amount of IFN- γ detected.

Activated effector γδ T cells have been shown to produce large amounts of IFN- γ. Intriguingly, γδ T cells have been shown to kill SARS in an IFN- γ dependent manner (88, 91).

γδ T cells seem to be heavily involved in children with COVID-19 (69). In early life, the innate immune system, including γδ T cells, is a predominant immune response (6). This is particularly interesting, considering that the severity of COVID-19 is significantly lower in children compared to adults (92).

While Carter et al. found a general lymphopenia in children, it seems to affect the γδ T cell population the most. Additionally, γδ T cells were the only activated T cell population, next to CD4 memory T cells.

This would indicate a strong involvement of γδ T cells in the immune response of COVID-19 in the most resistant population. While this needs to be further investigated, it seems that γδ T cells have great potential in aiding in combating the SARS-Cov-2 pandemic.

## γδ T Cells in Viral Infections

γδ T cells are involved in various types of viral infections [reviewed in Poccia et al. (93)]. For example, they seem to have antiviral activity against *human immunodeficiency virus* (HIV) (94, 95), which seems to be on par with that of CD8^+^ T cells (96). These cytolytic activities appear to be mostly through Vγ9Vδ2 T cells, however an inversion of the δ1 to δ2 ratio occurs in HIV patients (97, 98). Both subtypes might therefore be relevant in the immune response to HIV and other viruses (99).

Respiratory viruses, such as the influenza virus, also cause a γδ T cell response. The mRNA levels in mice post infection have shown high expression levels γδ TCR chain mRNA (100, 101). More recently activated human Vγ9Vδ2 T cells have been shown to effectively kill influenza infected human cells *in vitro* (102, 103). These γδ T cells have also been able to reduce severity and increase survival rates of immunodeficient, humanized mice infected with either human influenza (H1N1) or avian influenza (H5N1) (104).

Recently, it has been shown that the γδ T cell response to influenza changes throughout the human lifespan (105). While the main response was through IFN-γ producing Vγ9Vδ2 T cells, the γδ T cell repertoire was distinctly different in neonates and adults. The neonate γδ T cell repertoire showed a lot higher diversity, while the adult repertoire was heavily Vγ9Vδ2 dominant. Sant et al. further identified a vulnerability to influenza viruses in those neonate and elderly donors that lacked Vγ9Vδ2 TCRs. This shows that γδ T cells appear to be heavily involved in respiratory virus infections such as influenza viruses. Particularly striking is the importance of IFN- γ producing Vγ9Vδ2 T cells, which are commonly found in adult peripheral blood. Their importance in these different viral infections indicates a strong importance of this subtype in antiviral immunity.

There seems to be an even more direct link of γδ T cells antiviral capabilities against SARS-Cov-2. After the outbreak of SARS-Cov in 2003, Poccia et al. found an increase of γδ T cells in survivors 3 months after infection (88). More specifically, this increase was only observed in the Vδ2 subtype, which is most commonly found in blood. It was also shown in the same study, that Vγ9Vδ2 T cells isolated from blood were able to significantly reduce the viral load in *in vitro* studies.

These findings indicate an innate involvement of γδ T cells in viral infections. The immune response of these T cells to viral infections is further indication that they are involved in SARS-Cov-2 infections. Especially their connection to SARS infections seems promising. While there is still research, γδ T cells could become relevant for antiviral therapy, including COVID-19.

## γδ T Cells Against Cancer

γδ T cells are already used for medical purposes. Their innate response to inflammation and stressed cells has been used for cancer therapy. Their efficacy in this field could give indication for their use in other medical areas such as viral infections. This stands to reason as the γδ T cells, akin to NK-cells, may not substantially be able to distinguish between a virally infected and a transformed cancer forming cell.

Two different approaches have so far utilized γδ T cells in cancer therapy, reviewed by (10). γδ T cells are expanded in order to give the patient more cells to combat the cancer. In one treatment the γδ T cells are expanded *in vivo*. This is done by giving the patient zoledronate and IL-2 (106, 107). This method has shown to increase the survival rate of patients with multiple myeloma (108). However, this treatment has only had modest success in fighting cancer and has a drawback as IL-2 in low doses can promote immunosuppressive T_reg_ cells and can be toxic in high doses (109, 110).

Another treatment option is autologous adoptive transfer. In this method the γδ T cells are isolated from the patient and then expanded *ex vivo* (111). This method has been tested as a novel second-line therapy in various cancer types and has shown superior results in renal cell carcinoma over other established options (112–114).

In comparison, adaptive transfer has shown more success than the *in vivo* expansion treatment. However, it is also far more expensive and more time consuming since the γδ T cells need to be expanded over the course of 10-14 days in a specific purpose built laboratory (GMP facility).

When looking for γδ T cell therapy in other illnesses, both options should be considered.

Seeing the antiviral functions that γδ T cells have shown towards multiple viruses including SARS-Cov2, it might be worthwhile considering both alternatives for potential treatment.

## Conclusions

γδ T cells appear to be quite versatile in their immunological functions and their repertoire seems to be ever expanding. Especially the Vγ9Vδ2 subset has shown poly-cytotoxic functions in various fields. While the use of γδ T cells shouldn’t be limited to just this subset, their resourcefulness is promising.

When considering their use against COVID-19, two sides need to be weighed up. On the one side are the antiviral capacity that γδ T cells have shown. This is true for HIV and respiratory viruses such as influenza virus and now potentially SARS-Cov-2. However, on the other side is the risk of their pro-inflammatory activity adding to the cytokine storm which rages in COVID-19 patients. The γδ T cell-based treatments that have already been studied in a cancer setting could be a potential novel cure, but caution is highly advisable as a cytokine storm can have devastating effects and the potential negative effects of these treatments thus need to be assessed with great care.

Another matter to consider is the high variability of γδ T cells between individuals. As mentioned above, the γδ T cell population changes throughout the human lifespan. Additionally, individuals show different levels of subtypes and different absolute numbers in the same age groups.

Considering this, it seems more appropriate to look at potential cures on a case-by-case basis, rather than looking for a cure-all. A disease as complex as COVID-19 with varying degrees of cytokine release *via* cytokine storms and the highly polymorphic nature of the human immune system needs to be addressed according to these symptoms. We would strongly argue that γδ T cell based therapies could be treatments worth considering.

## Author Contributions

All authors listed have made a substantial, direct, and intellectual contribution to the work and approved it for publication.

## Funding

EPSRC, UK; Agency for Science Technology and Research, funding by A*STAR.

## Conflict of Interest

The authors declare that the research was conducted in the absence of any commercial or financial relationships that could be construed as a potential conflict of interest.

## Publisher’s Note

All claims expressed in this article are solely those of the authors and do not necessarily represent those of their affiliated organizations, or those of the publisher, the editors and the reviewers. Any product that may be evaluated in this article, or claim that may be made by its manufacturer, is not guaranteed or endorsed by the publisher.
